# Supplementary material for: Differential Early Mechanistic Frontal Lobe Responses to Choline Chloride and Soy Isoflavones in an Experimental Model of Fetal Alcohol Spectrum Disorder
Source: Int J Mol Sci. 2023 Apr 20;24(8):7595. doi: 10.3390/ijms24087595 (PMC10145811; doi:10.3390/ijms24087595)
Supplement: Supplementary file 1 [file ijms-24-07595-s001.zip › ijms-2276320-supplementary.pdf]

Supplementary Table S1: Immunoreactivities Measured by Duplex ELISA.

Abbreviation	Full Name	Gene Names	Product Functions
MAG1	Myelin Associated Glycoprotein	MAG; GMA	Myelin-associated cell-adhesion and signaling Type 1 transmembrane glycoprotein that maintains myelin-axon spacing by interacting with specific neuronal gangliosides, inhibits axonal regeneration and controls myelin formation. Minor component of myelin. (1, 2)
MOG	Myelin Oligodendrocyte Glycoprotein	MOG; MOG Ig-AluB; BTNL11; BTNL6	Transmembrane glycoprotein expressed on the oligodendrocyte cell surface and outer surface of myelin sheaths. Important for myelination. Expressed very late in development on oligodendrocytes and myelin sheaths. Surface marker of oligodendrocyte maturation with expression paralleling myelination. May regulate microtubular stability and function as an activator of complement (3)
MBP	Myelin Basic Protein	Myelin A1 Protein	This second most abundant protein in CNS myelin mediates adhesion and structural stability within compact myelin. MBP is a major component of myelin sheaths and is expressed in both oligodendrocytes and Schwann cells. MBP aids in the formation and stabilization of myelin membranes (4, 5).
PLP1	Proteolipid Protein 1	PLP, SPG2	The most abundant myelin membrane protein in the CNS, accounting for nearly 50% of the protein content. Transmembrane protein involved in multilamellar compact sheath stabilization, oligodendrocyte development and axonal survival. (6)
PDGFR-α	Platelet Derived Growth Factor Receptor, alpha polypeptide	CD140A, Alpha Platelet-Derived Growth Factor Receptor	Cell surface tyrosine protein kinase receptor. Developmentally expressed in embryonic and mature brains-neural progenitors, neurons, astrocytes, oligodendrocyte and vascular elements. Survival factor for oligodendrocyte progenitor cells. Also plays major roles in astrocyte metabolic activity, cell differentiation, and regulation of neurotransmitters. Mediates growth and maintenance of oligodendrocyte progenitor cells (OPCs) (7, 8)
GALC	Galactosylceramidase (galactocerebrosidase)	GALC; Galactocerebroside Beta-Galactosidase; GBD, DBP/GC, VDB	Galactosylceramide is the major type of glycosphingolipid in CNS myelin and consist of a ceramide with a galactose residue at the 1-hydroxyl moiety. Function in intracellular communication, cellular development, metabolized to sulfatide.

			GALC is a lysosomal protein that removes galactose from ceremide derivatives. (9)
GFAP	Glial Fibrillary Acidic Protein	GFAP; Intermediate Filament Protein	Astrocyte intermediate filament cytoskeletal protein. Responds to injury. A marker of acute CNS injury and neurological disorders. (10). Also roles in synaptic plasticity and regeneration (11); contributes to the development of vascular smooth muscle and endothelial cells (12)
UBQ	Ubiquitin	UBB Gene	Non-lysosomal, energy-dependent protein degradation machinery. Involved in post-translational modification of proteins, regulating protein abundance and turnover, including targeted removal of misfolded and aggregated unwanted proteins (13); mediates synaptic function
8-OHdG	8-Hydroxydeoxyguanosine	OGG1; 8-oxoguanine DNA Glycosylase	Major product of DNA oxidation. Generated due to ROS attack on guanine bases of DNA; 8-OHdG can lead to base-mismatch pairing G with T rather than C. Mostly known for oxidative stress, but paradoxically exogenous forms may relieve oxidative stress (14). During development marks impaired growth (15)
HNE	4-Hydroxy-2-nonenal	N/A	Major α,β-unsaturated aldehyde product of n-6 fatty acid oxidation and lipid peroxidation end product. HNE functions as a second messenger of oxidative/electrophilic stress, modulates cell survival/death via ER stress induction; promotes cell death via apoptosis as well. HNE reacts with many macromolecules containing histidine, cysteine and lysine residues leading to formation of HNE-protein adduction with attendant modification of activity. HNE mediated protein cross-linking induces carbonyl stress. (16)

1. Pronker MF, Lemstra S, Snijder J, Heck AJ, Thies-Weesie DM, Pasterkamp RJ, et al. Structural basis of myelin-associated glycoprotein adhesion and signalling. Nature communications. 2016;7:13584.
2. Quarles RH. Myelin-associated glycoprotein (MAG): past, present and beyond. Journal of neurochemistry. 2007;100(6):1431-48.
3. Johns TG, Bernard CC. The structure and function of myelin oligodendrocyte glycoprotein. Journal of neurochemistry. 1999;72(1):1-9.
4. Boggs JM. Myelin basic protein: a multifunctional protein. Cellular and molecular life sciences : CMLS. 2006;63(17):1945-61.
5. Stapulionis R, Oliveira CL, Gjelstrup MC, Pedersen JS, Hokland ME, Hoffmann SV, et al. Structural insight into the function of myelin basic protein as a ligand for integrin alpha M beta 2. J Immunol. 2008;180(6):3946-56.

6. Nadon NL, West M. Myelin proteolipid protein: function in myelin structure is distinct from its role in oligodendrocyte development. Developmental neuroscience. 1998;20(6):533-9.
7. Cardona HJ, Somasundaram A, Crabtree DM, Gadd SL, Becher OJ. Prenatal overexpression of platelet-derived growth factor receptor A results in central nervous system hypomyelination. Brain Behav. 2021;11(10):e2332.
8. Funa K, Sasahara M. The roles of PDGF in development and during neurogenesis in the normal and diseased nervous system. Journal of neuroimmune pharmacology : the official journal of the Society on NeuroImmune Pharmacology. 2014;9(2):168-81.
9. Ryckman AE, Brockhausen I, Walia JS. Metabolism of Glycosphingolipids and Their Role in the Pathophysiology of Lysosomal Storage Disorders. International journal of molecular sciences. 2020;21(18).
10. Yang Z, Wang KK. Glial fibrillary acidic protein: from intermediate filament assembly and gliosis to neurobiomarker. Trends in neurosciences. 2015;38(6):364-74.
11. Middeldorp J, Hol EM. GFAP in health and disease. Progress in neurobiology. 2011;93(3):421-43.
12. Osman I, Wang L, Hu G, Zheng Z, Zhou J. GFAP (Glial Fibrillary Acidic Protein)-Positive Progenitor Cells Contribute to the Development of Vascular Smooth Muscle Cells and Endothelial Cells-Brief Report. Arterioscler Thromb Vasc Biol. 2020;40(5):1231-8.
13. Turker F, Cook EK, Margolis SS. The proteasome and its role in the nervous system. Cell Chem Biol. 2021;28(7):903-17.
14. Ock CY, Kim EH, Choi DJ, Lee HJ, Hahm KB, Chung MH. 8-Hydroxydeoxyguanosine: not mere biomarker for oxidative stress, but remedy for oxidative stress-implicated gastrointestinal diseases. World J Gastroenterol. 2012;18(4):302-8.
15. Korkmaz KS, Debelec Butuner B, Roggenbuck D. Detection of 8-OHdG as a diagnostic biomarker. Journal of Laboratory and Precision Medicine. 2018;3:95-.
16. Dalleau S, Baradat M, Gueraud F, Huc L. Cell death and diseases related to oxidative stress: 4-hydroxynonenal (HNE) in the balance. Cell Death Differ. 2013;20(12):1615-30.
